# TAS2R16 Activation Suppresses LPS-Induced Cytokine Expression in Human Gingival Fibroblasts

**DOI:** 10.3389/fimmu.2021.726546

**Published:** 2021-12-15

**Authors:** Zhiyan Zhou, Ranhui Xi, Jiaxin Liu, Xian Peng, Lei Zhao, Xuedong Zhou, Jiyao Li, Xin Zheng, Xin Xu

**Affiliations:** ^1^ State Key Laboratory of Oral Diseases and National Clinical Research Center for Oral Diseases, West China Hospital of Stomatology, Sichuan University, Chengdu, China; ^2^ Department of Cariology and Endodontics, West China Hospital of Stomatology, Sichuan University, Chengdu, China; ^3^ Department of Periodontology, West China Hospital of Stomatology, Sichuan University, Chengdu, China

**Keywords:** human gingival fibroblasts (HGFs), taste receptor family 2 (TAS2Rs), cytokine, NF‐kappa B (NF-κB), cyclic AMP (cAMP)

## Abstract

Sustained and non-resolved inflammation is a characteristic of periodontitis. Upon acute inflammation, gingival fibroblasts release cytokines to recruit immune cells to counter environmental stimuli. The intricate regulation of pro-inflammatory signaling pathways, such as NF-κB, is necessary to maintain periodontal homeostasis. Nonetheless, how inflammation is resolved has not yet been elucidated. In this study, 22 subtypes of taste receptor family 2 (TAS2Rs), as well as the downstream machineries of Gα-gustducin and phospholipase C-β2 (PLCβ2), were identified in human gingival fibroblasts (HGFs). Various bitter agonists could induce an intensive cytosolic Ca^2+^ response in HGFs. More importantly, *TAS2R16* was expressed at a relatively high level, and its agonist, salicin, showed robust Ca^2+^ evocative effects in HGFs. Activation of TAS2R16 signaling by salicin inhibited the release of lipopolysaccharide (LPS)-induced pro-inflammatory cytokines, at least in part, by repressing LPS-induced intracellular cAMP elevation and NF-κB p65 nuclear translocation in HGFs. These findings indicate that TAS2Rs activation in HGFs may mediate endogenous pro-inflammation resolution by antagonizing NF-κB signaling, providing a novel paradigm and treatment target for the better management of periodontitis.

## Introduction

Periodontitis is a chronic inflammatory disease that is triggered by the accumulation of dental plaques. It involves a severe chronic inflammation that destroys tooth-supporting tissues, and if left untreated, it leads to tooth loss (1). Periodontitis is the primary cause of tooth loss in adults. It not only affects oral health but also has a close relationship with systemic diseases, such as diabetes mellitus, cardiovascular diseases, rheumatoid arthritis, etc (2). Gingival fibroblasts are the predominant cells in gingival connective tissues, contributing to sustained inflammation in periodontal diseases (3, 4). Under inflammatory conditions, human gingival fibroblasts (HGFs) function as accessory immune cells, and together with other cells such as gingival epithelial cells, secreting pro-inflammatory cytokines and promoting neutrophils homing (5, 6). Nonetheless, sustained release of cytokines and immune cell recruitment will cause non-resolved inflammation, thus contributing to inflammatory tissue breakdown and the development of periodontitis. The regulation of cytokine release by fibroblasts during inflammation is mainly mediated by NF-κB signaling (7), while the mechanisms of periodontal inflammation resolution remain poorly understood.

Taste receptor family 2 (TAS2Rs), which belong to the G-protein-coupled receptor family, are initially identified in taste buds and function as peripheral taste receptors for bitter stimuli (8, 9). Recent studies have identified the expression of TAS2Rs in a variety of extra-gustatory tissues, including the respiratory tract, gastrointestinal mucosa, urethra, heart, and gingiva (10–14). The *TAS2Rs*-expressing cells that reside in these tissues also express several downstream machineries that are essential for the taste signaling cascade in type II taste cells, such as G protein subunit Gα-gustducin, phospholipase C-β2 (PLCβ2), and transient receptor potential cation channel melanostatin (TRPM) 5 (15–17). Most studies have demonstrated that extra-gustatory TAS2Rs function as immune sentinels in mammalian innate immune responses to environmental stimuli, including metabolites derived from bacteria or parasites (18–21). In addition, intracellular Ca^2+^ elevation is involved in TAS2Rs-mediated downstream effects, such as antimicrobial peptide (AMP) secretion, pathogen clearance, and respiratory reflexes (18, 22). Of note, TAS2Rs activation can antagonize lipopolysaccharide (LPS)-induced inflammatory mediator production both in human whole blood and lung macrophages (23, 24), suggesting the potential role of TAS2Rs in the tight control of inflammation. However, the mechanisms and biological significance of TAS2Rs-induced anti-inflammatory effects have not been well documented. Our recent study revealed that bitter tastant denatonium benzoate could alleviate periodontitis in a TAS2Rs-dependent manner in mice and confirmed that gingival solitary chemosensory cells (gSCCs) contributed to such an effect by mediating AMP expression (25).

Given the wide expression of TAS2Rs in various types of cells, we hypothesize that gingival fibroblasts express TAS2Rs, which are involved in the attenuation of excessive inflammatory responses and contribute to the tight regulation of periodontal inflammation. In this study, we confirmed the expression of *TAS2Rs* and downstream signaling components in HGFs. Activation of TAS2R16 by salicin antagonized LPS-induced cytokine expression by downregulating intracellular cAMP and inhibiting the NF-κB signaling cascade in HGFs.

## Materials and Methods

### Primary Human Gingival Fibroblasts Culture

The current study was approved and supported by the Institution Review Board of West China Hospital of Stomatology, Sichuan University (WCHSIRB-OT-2020-049). The experiment was in accordance with the Declaration of Helsinki principles. Gingival tissues were collected from healthy volunteers (n=4, aged between 18~30 years old) who experienced the third molar extraction with no clinical symptoms of either oral or systematic diseases. Tissues were immersed into dulbecco’s modified eagle medium (DMEM; Gibco, New York, NY, USA) with 10% fetal bovine serum (FBS; Gibco, New York, NY, USA) and 1% penicillin-streptomycin solution (Hyclone, Logan, UT, USA) on ice and quickly transferred to the lab. The pooled tissues were rinsed by Hank’s Balanced Salt Solution (HBSS, Hyclone, Logan, UT, USA) with 1% penicillin-streptomycin, torn off epithelium, cut into small pieces, and then incubated with 2 mM type I collagenase (Sigma-Aldrich, Steinheim, Germany) at 37°C for 60 min. After centrifugation (200 g, 3 min), cells were resuspended in DMEM and then seeded on a T25 culture flask (Corning, New York, NY, USA), and cultured in the humidified atmosphere at 37°C with 5% CO_2_. P3-P8 of the cells were used for further experiments.

### RNA Extraction and Reverse Transcription Polymerase Chain Reaction

Total RNA was extracted from fresh gingival tissues (n=8) and cultured HGFs (n=4) with TRIzol regent (Life Technologies, Carlsbad, CA, USA) and MiniBest Universal RNA Extraction Kit (TAKARA, Tokyo, Japan), respectively, according to the manufacturer’s instructions. The precipitate was air-dried and resuspended in RNase-free water. The purity and concentration of isolated RNA were determined by a nanodrop 2000 spectrophotometer (Thermo Fisher Scientific, Waltham, MA, USA). cDNA was generated from a total of 1 µg RNA using RT reagent Kit with gDNA Eraser (TAKARA, Tokyo, Japan). Aliquot without reverse transcriptase was prepared as the negative control. PCR was performed using PrimeSTAR^®^ Max DNA Polymerase (TAKARA, Tokyo, Japan) to detect the existence of *TAS2Rs* and downstream molecules (primers listed in **Supplementary Table 1**). Besides, quantitative real-time PCR was performed to determine the relative expression level of *TAS2Rs* in HGFs (primers listed in **Supplementary Table 1**). The expression level of target genes normalized with glyceraldehyde-3-phosphate dehydrogenase (*GAPDH*) was calculated by 2^-ΔΔCT^ method.

### Immunofluorescence Staining

Cultured HGFs were fixed in 4% formaldehyde for 15 min, followed by permeabilization with 0.2% Triton X-100 for 15 min at room temperature. After blocked with 5% goat or rabbit serum for 60 min at room temperature, cells were incubated at 4°C overnight with the desired primary antibodies for vimentin (1 µg/mL; Cat. No. ab8978 Cambridge, UK), Gα-gustducin (1:100; Cat. No. PA5-23986, Invitrogen, Carlsbad, CA, USA), PLCβ2 (1:100; Cat. No. OAAB05844, Aviva Systems Biology, San Diego, CA, USA), or phosphos-p65 ser276 (1:100; Cat. No. EPR17622, Abcam, Cambridge, UK). After washed 3× for 5 min with PBS, cells were incubated for 1 h at room temperature with species-specific secondary antibodies conjugated with different fluorophores (1:200; Cat. No. ab150076, Abcam. 1:200; Cat. No. L30113, SAB, Nanjing, China). DAPI (Solarbio, Beijing, China) in deionized water was used to visualize the nuclei. Images were taken by an inverted confocal laser scanning microscope (CLSM) (Olympus, Tokyo, Japan) equipped with an Ar laser (488 nm) and LED laser (559 nm). The quantitative analysis was performed by IMARIS software (Bitplane, Zurich, Switzerland).

### TAS2R16 Transfection

TAS2R16-pcDNA™3.1/Zeo^(+)^ and coupling chimeric G protein Gα16-gust44 construct (kindly provided by Peihua Jiang, Monell Chemical Senses Center) were transiently transfected into ~90% confluency HEK293 cells maintained in 96-well plate using lipofectamine 2000 (0.5 μL/well; Thermo Fisher Scientific, Waltham, MA, USA) as described previously (25). 24 h after transfection, the calcium flux assay was performed.

### Calcium Mobilization Assay

HGFs were seeded (1×10^4^ cells per well) into a black 96-well plate with clear bottoms (Corning, New York, NY, USA) and cultured overnight. Cells were washed twice with Dulbecco’s Phosphate Buffered Saline (DPBS; Hyclone, Logan, UT, USA) containing 100 mg/L CaCl_2_ and 100 mg/L MgCl_2_·6H_2_O and then loaded with Fluo-4 AM (2.5 ng/µL; Thermo Fisher Scientific, Waltham, MA, USA) and F-127 (0.5 ng/µL; Life Technologies, Carlsbad, CA, USA) for 1 h at room temperature in the dark. After three washes with DPBS, cells were incubated in the dark for another 30 min for complete de-esterification of the dye. Cells were pre-treated with probenecid (1 mM; MedChemExpress, Monmouth Junction, NJ, USA) for 1 h, U73122 (10 µM; MCE, Monmouth Junction, NJ, USA) for 1 h, or AITC (1 mM; Sigma, Steinheim, Germany) for 30 min before tastants stimulation (26–28). Bitter components used in this study and their known bitter taste receptors were listed in **Supplementary Table 2**.

Calcium mobilization traces were recorded using Flexstation III (Molecular Devices, Sunnyvale, CA, USA). Relative fluorescence units (excitation at 488 nm, emission at 525 nm, and cutoff at 515 nm) were read every 2 s for a duration of 200 s (bitter compounds were added at 30 s). For calcium imaging, cells were examined under an Olympus IX-83 P2ZF microscope with a standard GFP filter. Images were acquired every 1 s for 140 s (bitter compounds were injected into the wells at ~10 s). 10 µM ATP (Solarbio, Beijing, China) and DPBS were used as positive and negative controls, respectively. Curves of fluorescence intensity normalized by baseline (F/F_0_; F_0_ was determined as the average of the first 5 readings) to time were illustrated with GraphPad 8.0 (GraphPad Software, San Diego, CA, USA).

### *In Vitro* LPS-Induced Inflammation Model

HGFs were grown into ~50% confluency and then transfected with 50 nM TAS2R16-siRNA or non-target-siRNA (listed in **Supplementary Table 1**; synthesized by Hippo biotechnology) using lipofectamine 2000 according to the manufacturer’s instruction. *TAS2R16* silencing efficiency was confirmed by RT-qPCR. The transfected cells were cultured till ~90% confluency (for 48 h) and treated with LPS (5 µg/mL; Cat. No. L2880, Sigma, Steinheim, Germany) and (or) D (-)-salicin (2 mM; Cat. No. S0625, Sigma, Steinheim, Germany) for 24 h. In other experiments, HGFs were grown into ~80% confluency. LPS (5 µg/mL for all assays), salicin (2 mM for all assays except for the cell proliferation assay and the calcium mobilization assay), U73122 (1 µM for RT-qPCR, and 0.5 µM for Western Blot), and forskolin (5 µM for RT-qPCR and Western Blot) were alone or combinatorically applicated to HGFs for 24 h. Total RNA was extracted using MiniBest Universal RNA Extraction Kit. Inflammatory cytokines were quantified by real-time quantitative RT-PCR (primers listed in **Supplementary Table 1**). The phosphorylation of NF-κB was evaluated by western blotting and CLSM. Additionally, culture supernatant or cell lysate was collected for subsequent experiments as described below.

### Cell Proliferation

HGFs were seeded at a density of 1×10^4^ onto a 96-well plate and incubated for 24 h. The cells were treated with an increasing concentration of salicin (range from 0.1 to 10 mM). After 24 h, the medium was changed and the mixture of Cell Counting Kit-8 (CCK-8) solution (DMEM: CCK-8 = 9:1; ApexBio, Houston, Texas, USA) was supplemented to every well for 1.5 h at 37°C when the optical density (OD) at 450 nm of non-treated HGFs is about 1.

### Neutrophil Isolation and Cell Migration Assay

Human peripheral blood was collected from consented healthy volunteers. Neutrophils were isolated from EDTA-anticoagulated whole blood by Percoll density centrifugation using 2 discontinuous gradients (1.075 and 1.090) followed by erythrocyte lysis as previously described (29). In brief, 2 mL 60% Percoll solution (Sigma, Steinheim, Germany) was layered over 2 mL 75% Percoll solution in the FBS-pretreated tube. 2 mL peripheral blood was centrifugated and suctioned for leukocytic cream, which was carefully layered over 60% Percoll. The suspended substance between the two layers was extracted, and residual red cells were lysed by Red Cell Lysis Buffer (Biosharp, Hefei, China). Cells were suspended in Roswell Park Memorial Institute (RPMI) 1640 medium (Gibco, New York, NY, USA) at a density of 1×10^6^ cells/mL. Cell viability and purity (typically >98%) were determined by Trypan blue exclusion (Solarbio, Beijing, China) and Wright’s-Giemsa staining solution (Solarbio, Beijing, China), respectively. Chemotaxis assay was conducted as previously reported with minor modification using the transwell system (Cat# 3422, Corning, New York, NY, USA) (30). Culture supernatant of HGFs treated with LPS and (or) D (-)-salicin was collected and filtered. 100 µL cells were seeded into upper chambers, and 600 µl supernatant was added to lower chambers. After 2 h incubation (37°C, 5% CO_2_), 500 µl aliquot in the lower chambers was harvested to a 96-well plate. Randomly selected fields in each well were recorded by an Olympus invert microscope. The migrated neutrophils were counted by ImageJ (National Institutes of Health, Bethesda, MD, USA).

### Western Blot

HGFs treated with LPS, and (or) D (-)-salicin were scraped from wells and washed with 4°C PBS. Total protein was extracted using cell lysis buffer (Beyotime Biotechnology, Shanghai, China) supplemented with 1% protease inhibitor cocktail (Beyotime Biotechnology, Shanghai, China) according to the manufacturer’s instruction. The equivalent amount of protein was loaded and separated by sodium dodecyl sulfate poly-acrylamide gel electrophoresis (SDS-PAGE) in 10% gels. The gels were transferred to polyvinylidene difluoride (PVDF) membrane for 1 h. The membranes were blocked with 5% non-fat powdered milk (Sangon Biotech, Shanghai, China) for 1.5 h at room temperature and then incubated with desired primary antibodies for p65 (1:1000; Cat. No. ER0815, Huabio, Hangzhou, China), p-p65 ser276 (1:1000), or histone-H3 (1:1000; Cat. No. 17168-1-AP, Proteintech, Wuhan, China) overnight at 4°C. After that, the membranes were washed 3× for 10 min with 1× Tris Buffered Saline Tween (TBST), incubated with goat anti-rabbit IgG (HPR) secondary antibody (1:5000; Cat. No. ab6721, Abcam, Cambridge, UK) for 1 h, and then washed 6× for 10 min with 1× TBST. The proteins bands were visualized by ChemiDoc MP Imaging System (Bio-Rad, Hercules, CA, USA) and quantified by ImageJ software.

### IL-8 and Intracellular cAMP Quantification

HGFs treated with LPS and (or) D (-)-salicin were centrifugated for 10 min at 4°C. The supernatant was collected for IL-8 quantification (Zci Bio, Shanghai, China), and the cell lysate was prepared by Ultrasonic Cell Disruption System (QIAGEN, Germantown, Germany) to perform cAMP quantification (Zci Bio, Shanghai, China) according to the manufacturer’s instructions, respectively. The results were collected by Flexstation III (OD560), analyzed using the generated standard curves, and illustrated as nmol/L.

### Statistical Analysis

Each *in vitro* experiment was repeated separately three times. Statistical analysis was performed using GraphPad 8.0. Data were presented as the mean ± standard deviation. Comparisons between different groups were performed by one-way ANOVA followed by Tukey’s multiple comparisons test or unpaired t-test in different contexts. Differences were considered significant when the two-tailed *P* value was <0.05.

## Results

### Primary HGFs Express Multiple TAS2Rs and Downstream Machineries

**Figure 1:**
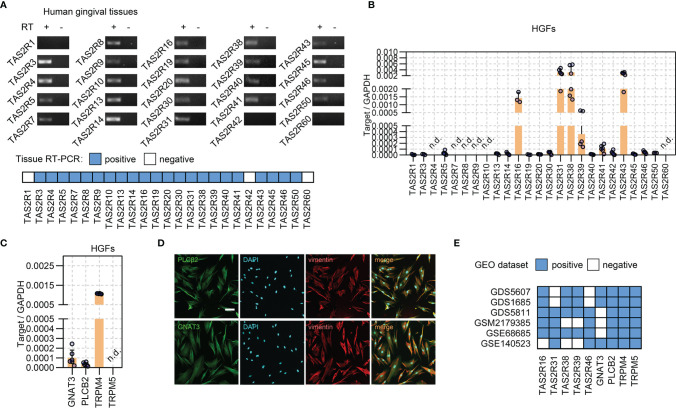
Expressions of *TAS2Rs* and downstream signaling components in HGFs. **(A)** Expression of *TAS2Rs* in human gingival tissues (pooled from 8 volunteers) examined by RT-PCR. RT +/−: with/without reverse transcription. Expression levels of *TAS2Rs* **(B)** and downstream signaling components **(C)** in primary HGFs determined by qPCR. Data are present as mean ± standard deviation (s.d.). Each circle in the bar represents an individual replicate. n.d., not detected. **(D)** Immunofluorescence staining of HGFs with vimentin (red) and PLCβ2 (green), or vimentin (red) and Gα-Gustducin (green), respectively. Nuclei are stained by DAPI (blue). Scale bar, 100 μm. **(E)** GEO data analysis for *TAS2Rs* and key signaling elements expression in HGFs.

Fresh gingival tissues were collected to determine the expression of *TAS2Rs*. RT-PCR revealed the expression of 22 subtypes of *TAS2Rs* **(** **Figure 1A** and **Supplementary Figure 1** **)**. As fibroblasts are predominant in gingival connective tissue and are closely involved in the inflammatory responses to environmental stimuli (5, 31, 32), we investigated the expression of *TAS2Rs* in HGFs. In the cultured primary HGFs (pooled from four gingival samples), *TAS2R16*, *TAS2R31*, *TAS2R38*, *TAS2R39*, and *TAS2R43* were the most expressed among the 19 *TAS2Rs* detected in HGFs **(** **Figure 1B** **)**. Meanwhile, the expression of *TAS2R4*, *TAS2R7*, *TAS2R8*, *TAS2R9*, *TAS2R10*, and *TAS2R60* was not detected **(** **Figure 1B** **)** in the samples. In addition to taste receptors, taste signaling elements Gα-gustducin (*GNAT3*) and PLCβ2 (*PLCB2*) were identified by real-time quantitative RT-PCR **(** **Figure 1C** **)** and immunofluorescence staining **(** **Figure 1D** **)** in cultured HGFs. Moreover, *TRPM4*, instead of *TRPM5*, was detected in HGFs **(** **Figure 1C** **)**. To further confirm the expression of *TAS2Rs* and downstream machinery in HGFs, we analyzed several datasets containing HGF expression profiles from the Gene Expression Omnibus. *TRPM4*, *TRPM5*, *PLCB2*, and a variety of *TAS2Rs*, including *TAS2R16*, *TAS2R31*, *TAS2R38*, *TAS2R39*, and *TAS2R43*, were identified in HGFs in most of the selected datasets **(** **Figure 1E** and **Supplementary Table 3** **)**. Of note, the expression levels of *TRPM5* in these datasets were significantly lower relative to *TRPM4*, particularly in datasets GSE140523, in which only one out of the five samples expressed *TRPM5* (**Supplementary Table 4**). The expression of *GNAT3* was reported in three datasets **(** **Figure 1E** and **Supplementary Tables 3**, **4** **)**.

### Salicin Activates TAS2R16 in Primary HGFs

**Figure 2:**
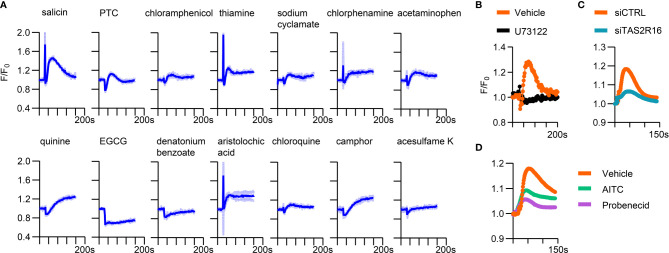
A variety of bitter tastants could induce intracellular calcium elevation in HGFs. **(A)** Calcium response curves of HGFs to bitter tastants. 10 mM D (-)-salicin, 500 µM PTC, 1 mM chloramphenicol, 1 mM thiamine, 10 mM sodium cyclamate, 100 µM chlorphenmine, 3 mM acetaminophen, 20 µM quinine, 500 µM epigallocatechin gallate (EGCG), 10 mM denatonium benzoate, 10 µM aristolochic acid, 5 mM chloroquine, 1 mM camphor and 10 µM acesulfame K were used as stimulus. Data are presented as mean (dark blue line) ± s.d. (light blue shadow), n=3 independent experiments. Representative calcium response curves of **(B)** 10 µM U73122 pre-treated HGFs, **(C)** siCTRL/siTAS2R16 HGFs, and **(D)** 1mM AITC or 1 mM probenecid pre-treated HGFs to 2 mM salicin. siCTRL: non-target control for siRNA silencing; siTAS2R16: TAS2R16 knockdown by siRNA silencing; AITC, allylisothiocyanate.

To further confirm the function of *TAS2Rs* expressed in HGFs, we screened candidate bitter tastants that could induce intracellular calcium accumulation in HGFs. Several agonists evoked increased intracellular Ca^2+^ levels in primary HGFs, including salicin, quinine, phenylthiocarbamide (PTC), aristolochic acid, camphor, and thiamine. In addition, low-level but sustained Ca^2+^ increment was observed when stimulating HGFs with sodium cyclamate, chloroquine, chlorphenamine, and chloramphenicol **(** **Figure 2A** **)**. Salicin induced the most robust Ca^2+^ response among the tested agents, and TAS2R16, the specific receptor for salicin **(** **Supplementary Figure 2** **)** (26, 33), showed a relatively high expression level in HGFs. We focused on the physiological function of TAS2R16 activation in the following experiments.

Pretreatment with the PLC inhibitor U73122 (22) and *TAS2R16* knockdown abolished the calcium response of HGFs to salicin **(** **Figures 2B, C** **)**, confirming that the intracellular calcium-boosting effect of salicin was dependent on TAS2R16 and downstream machineries such as PLCβ2. Additionally, the TAS2R16/TAS2R38 inhibitor probenecid (26) and non-specific TAS2Rs inhibitor allylisothiocyanate (AITC) (20) repressed the salicin-induced calcium response in HGFs **(** **Figure 2D** **)**, further suggesting that salicin induces intracellular calcium accumulation in HGFs *via* TAS2R16.

### TAS2R16 Activation Inhibits LPS-Induced Cytokine Expression in HGFs

**Figure 3:**
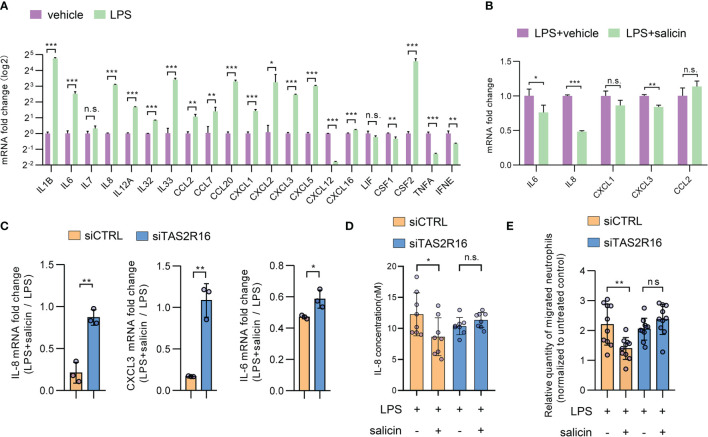
TAS2R16 activation inhibits lipopolysaccharide-induced cytokine expression. **(A)** mRNA expression level of pro-inflammatory cytokines in HGFs treated with LPS or vehicle. The expression level of each target is normalized with *GAPDH*. **(B)** mRNA expression level of IL-6, IL-8, CXCL1, CXCL3 and CCL2 in HGFs treated with LPS alone or LPS and salicin. **(C)** Fold changes of mRNA expression level are calculated by normalizing the expression level to HGFs treated with LPS alone. Results are presented as fold change in LPS+salicin treated group relative to LPS treated group. Each circle represents an independent sample (n=3). **(D)** IL-8 levels in the supernatant of siCTRL and siTAS2R16 HGFs treated with salicin and LPS. Each circle represents a datum from one well (n=8). **(E)** Neutrophil migration ability measured by the transwell assay. Relative quantities of migrated neutrophils in each group are calculated by normalizing the untreated group. Each circle represents a datum from one field (n=10). LPS 5 µg/mL and salicin 2 mM in each figure. Data are presented as mean ± s.d. Comparisons between different groups in **(D, E)** were performed by one-way ANOVA followed by Tukey’s multiple comparisons test. In **(A–C)**, the comparisons were performed by unpaired t-test. *p < 0.05; **p < 0.01; ***p < 0.001, n.s., not significant.

HGFs secrete multiple cytokines in the gingival tissues of patients with periodontitis (32). Upon acute inflammation, pathogen-associated molecular patterns, such as LPS, induce cytokine expression in gingival fibroblasts (34). We investigated whether TAS2R16 activation impacted cytokine expression in HGFs in the context of LPS-induced inflammation. Salicin (0-2 mM) did not significantly repress the cell viability of HGFs **(** **Supplementary Figure 3** **)**. Under the normal condition, cytokine expression of HGFs was maintained at a relatively low level and was unchanged after salicin treatment **(** **Supplementary Figure 4** **)**. In addition, *TAS2R16* silencing also did not affect the expression level of selected cytokines **(** **Supplementary Figure 5** **)**, implying that TAS2R16 function and our siRNA known-down manipulation had no effects on cytokine expression in HGFs at baseline. Twenty-two candidate cytokines or chemokines were screened based on their expression levels in HGFs or their possible relationship with periodontal inflammation. As expected, LPS treatment significantly induced cytokine expression in HGFs **(** **Figure 3A** **)**. The top five most expressed cytokines after LPS treatment including IL-6, IL-8, CXCL1, CXCL3, and CCL2 (**Supplementary Table 5**) were selected for further investigation. Simultaneous treatment with salicin significantly inhibited the LPS-induced expression of IL-6, IL-8, and CXCL3, rather than CXCL1 and CCL2 **(** **Figure 3B** **)**. Consistently, *TAS2R16-*silencing diminished the inhibitory effect of salicin on the expression of IL-6, IL-8, and CXCL3 in HGFs **(** **Figure 3C** **)**, and promoted IL-8 secretion that was suppressed by salicin in the supernatant of HGF-cultures **(** **Figure 3D** **)**, further suggesting that salicin can inhibit LPS-induced cytokine expression in HGFs in a TAS2R16-dependent manner.

IL-8, also known as CXCL8, is closely related to neutrophil recruitment during periodontal inflammation (35, 36). Transwell assay revealed that the conditioned medium from LPS-treated HGFs significantly enhanced neutrophil migration, while simultaneous treatment with salicin significantly repressed this effect **(** **Figure 3E** **)**. Furthermore, treatment with salicin did not alter the ability of the conditioned medium to attract neutrophils when *TAS2R16* was silenced in HGFs **(** **Figure 3E** **)**.

### TAS2R16 Activation Represses LPS-Induced Intracellular cAMP Accumulation and NF-κB Nuclear Translocation

**Figure 4:**
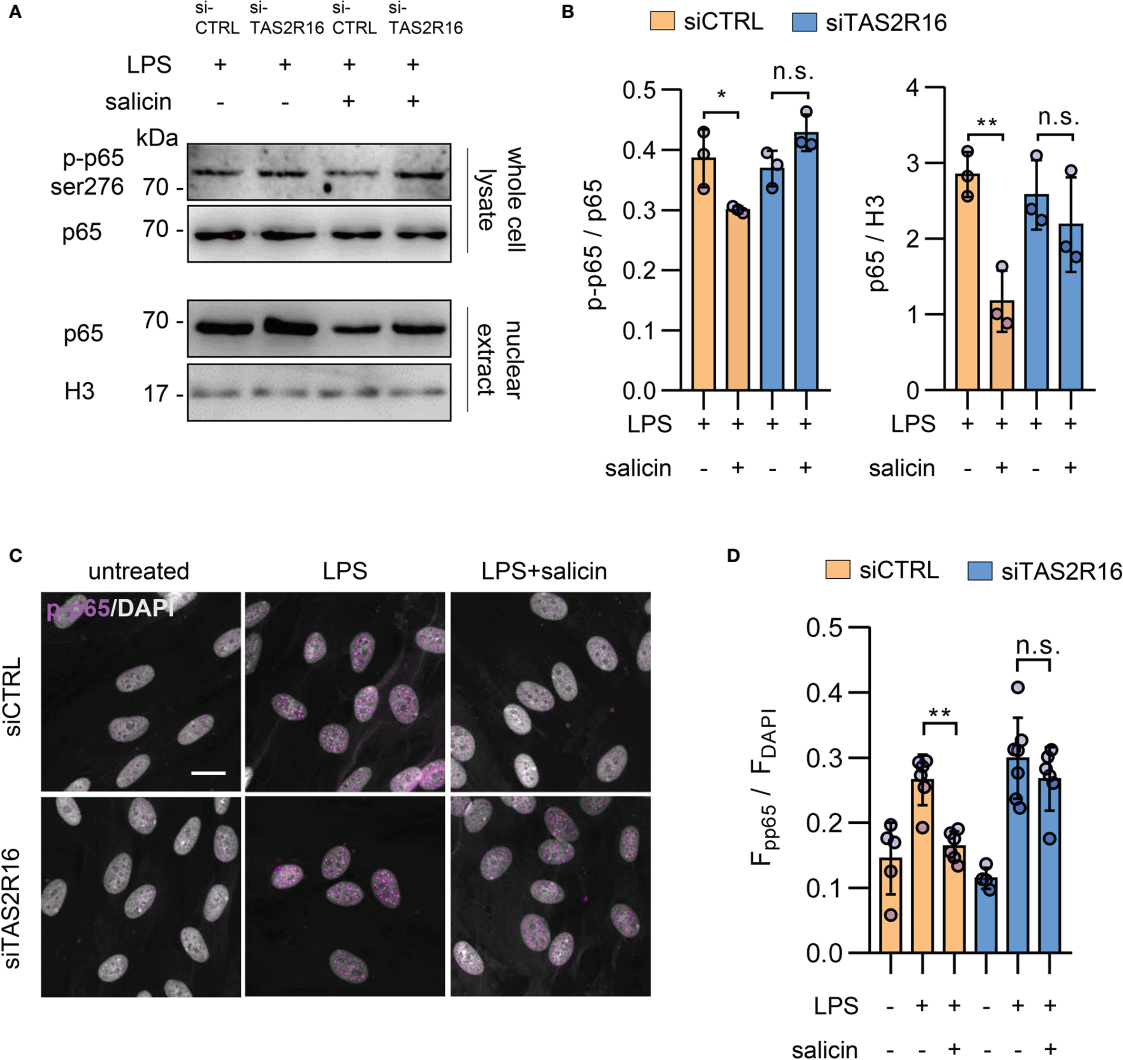
TAS2R16 activation represses LPS-induced NF-κB phosphorylation and nuclear translocation. **(A)** Representative Western blot images for p65 phosphorylation and nuclear translocation from siCTRL and siTAS2R16 HGFs treated with LPS or LPS+salicin (p65 for whole cell lysate and H3 for nuclear extract). **(B)** Bar graph depicts the normalized ratio of p65 to histone H3 and p65-ser^276^ to p65. Each circle represents an independent experiment (n=3). **(C)** Representative images of immunofluorescence staining for phosphorylated p65 (p-p65, in magenta color) in HGFs. Nuclei are stained by DAPI (gray). Scale bar, 20 μm. **(D)** Quantitative analysis for p-p65 and DAPI co-localization is presented. Each circle represents a datum from one field (n=4-7). LPS 5 µg/mL and salicin 2 mM in each figure. Data are presented as mean ± s.d. Comparisons between different groups were performed by one-way ANOVA followed by Tukey’s multiple comparisons test. *p < 0.05; **p < 0.01; n.s., not significant.

The expression of IL-8 is regulated by multiple transcriptional factors, of which NF-κB plays a key role (31). In cultured primary HGFs, salicin treatment significantly repressed the LPS-induced phosphorylation of p65 at ser^276^, as well as the level of p65 in nuclear extracts, in a TAS2R16-dependent manner **(** **Figures 4A-D** **)**, implying that TAS2R16 activation countered LPS-induced cytokine expression *via* negatively regulating NF-κB signaling activation in HGFs.

**Figure 5:**
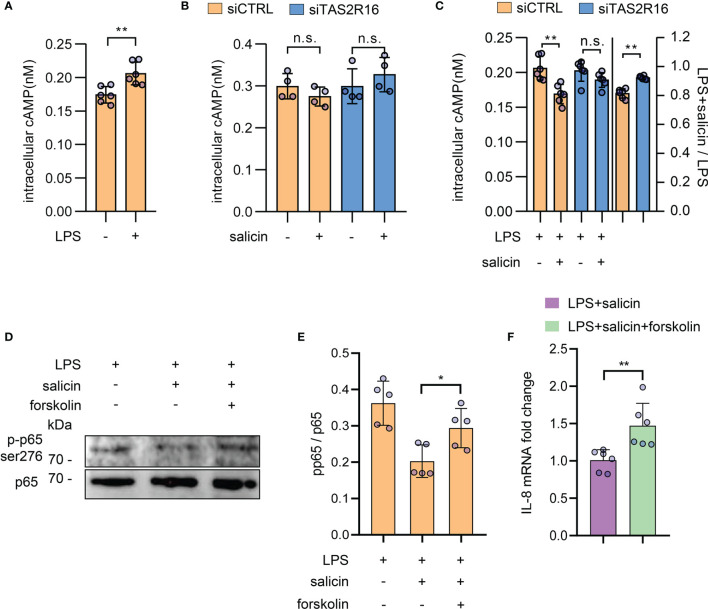
TAS2R16 activation represses LPS-induced intracellular cAMP accumulation. **(A)** Intracellular cAMP levels of HGF treated with LPS or vehicle. Each circle represents a datum from one well (n=6). **(B)** Intracellular cAMP levels of siCTRL and siTAS2R16 HGFs treated with salicin or vehicle control in the absence of LPS. Each circle represents a datum from one well (n=4). **(C)** In the left panel, data are presented as intracellular cAMP levels of siCTRL and siTAS2R16 HGFs treated with LPS or LPS+salicin. In the right panel, results are presented as fold change of cAMP levels in LPS+salicin treated group relative to LPS treated group. Each circle represents a datum from one well (n=6). **(D)** Representative Western blot images for p65 phosphorylation in HGFs treated with LPS, salicin, and forskolin. **(E)** The normalized ratio of p65-ser^276^ to p65. Each circle represents an independent experiment (n=5). **(F)** mRNA expression level of IL-8 in HGFs treated with LPS, salicin and forskolin. Each circle represents a sample. LPS 5 µg/mL, salicin 2 mM, and forskolin 5 µM in each figure. Data are presented as mean ± s.d. Comparisons between different groups in **(B, E)**, and the left panel in **(C)** were performed by one-way ANOVA followed by Tukey’s multiple comparisons test. In **(A, F)**, and the right panal in **(C)**, the comparisons were performed by unpaired t-test. *p < 0.05; **p < 0.01; n.s., not significant.

It has been reported that cAMP is related to the phosphorylation of NF-κB p65 (37, 38), which functions as a central downstream effector in LPS-induced inflammation (7, 39). We confirmed the increased intracellular cAMP level in HGFs induced by LPS **(** **Figure 5A** **)**. cAMP is also one of the well-documented downstream effectors of taste receptor signaling. TAS2R activation can decrease the cAMP level. We, therefore, speculated that TAS2R16 activation might inhibit the LPS-induced p65 phosphorylation *via* downregulation of intracellular cAMP. Although salicin treatment alone had no significant effect on the intracellular level of cAMP in HGFs **(** **Figure 5B** **)**, it decreased the LPS-induced cAMP accumulation by ~20%, an effect that could be abolished by siRNA knock-down of *TAS2R16* **(** **Figure 5C** **)**. Of note, stimulation of cAMP synthesis in HGFs by treatment of adenylate cyclase (AC) activator forskolin, weakened the inhibitory effects of salicin on p65 ser^276^ phosphorylation **(** **Figures 5D, E** **),** and rescued LPS-induced IL-8 expression that was repressed by salicin **(** **Figures 5F** **)**, further demonstrating that cAMP was involved in the inhibitory effects of TAS2R16 signaling against LPS-induced inflammation.

Another well-known downstream effect of TAS2Rs activation is the elevation of intracellular Ca^2+^ in a PLCβ2-dependent way, which was confirmed in our cultured HGFs **(** **Figures 2A, B** **)**. Several studies reported that LPS-induced cytokine expression was also partly dependent on intracellular calcium elevation mediated by PLC (40, 41). Consistently, we also found largely abrogated expression of IL-8, IL-6, and CXCL3 in the treatment of U73122 under the LPS-induced inflammatory condition **(** **Supplementary Figure 6A** **)**. In addition, U73122 treatment did not mitigate the inhibitory effect of salicin on LPS-induced expression of IL-8 and CXCL3, as well as p65 nuclear translocation **(** **Supplementary Figures 6B, C** **)**, suggesting that salicin is less likely to antagonize LPS-induced cytokine expression and NF-κB activation *via* Ca^2+^.

## Discussion

TAS2Rs and their downstream signaling molecules have been identified in multiple extra-gustatory tissues throughout the body, including the respiratory tract, gastrointestinal mucosa, and heart. Our recent study also identified a specific group of *TAS2R*-expressing cells in the gingiva of mice, defined as gingival solitary chemosensory cells (25). HGFs, the predominant cell type in gingival connective tissues, together with other cells like gingival epithelial cells, play an essential role in the maintenance of periodontal homeostasis. However, HGF-originated *TAS2Rs* have barely been investigated. Here, we found that HGFs expressed various *TAS2Rs* and downstream machineries, including *TAS2R16*, *TAS2R38*, *TAS2R31*, *TAS2R39*, *TAS2R43*, and *TRPM4*, and responded to a diverse range of bitter tastants, particularly salicin. TRPM4, like TRPM5, is a voltage-sensitive and monovalent-selective channel activated by oscillatory changes in intracellular Ca^2+^ (42). A recent study revealed the critical role of TRPM4 in response to bitter, sweet, and umami stimuli (43). TRPM4 activation can increase initial cell depolarization, activate the continuous release of ATP, and thus mediate taste responses (43). The current study identified *TRPM4* instead of *TRPM5* in HGF samples. In the selected datasets, the expression levels of *TRPM5* in HGFs were also significantly lower in comparison with that of *TRPM4*, especially in GSE140523, in which only one out of the five samples expressed *TRPM5* (44). Nonetheless, the discrepancy between our RT-qPCR data and GEO RNA-seq data regarding the *TRPM5* expression in HGFs may be caused by different sampling and detection methods. Additionally, we detected higher expression levels of *TAS2R16*, *TAS2R38*, *TAS2R31*, *TAS2R39*, and *TAS2R43* in primary HGFs. TAS2R43 is responsive to a diverse constellation of structurally different compounds and has been identified in human airway epithelia (45, 46). Denatonium benzoate, a bitter agonist of TAS2R43 and other TAS2Rs, can stimulate airway epithelial ciliary motility *via* taste signaling (45). *TAS2R39* is highly expressed in human airway smooth muscle cells and human monocyte-derived macrophages (6, 22). Increased phagocytosis can be induced by the TAS2R14/TAS2R39 agonists apigenin and chrysin (6). *TAS2R38* encodes the bitter taste receptor in humans, which preferentially recognizes PTC. Clinical studies have revealed that gene polymorphisms of *TAS2R38* are correlated with protective effects against caries (47, 48). In addition, TAS2R38 appears to be an essential mediator of sinonasal epithelial defense and is responsible for respiratory bacterial infections (18). *TAS2R16* has been identified in human neuronal tissue. Salicin, a specific agonist of TAS2R16, may modulate neurite outgrowth *via* TAS2R16 activation (49). Given the relatively higher expression of *TAS2R16*, *TAS2R38*, *TAS2R31*, *TAS2R39*, and *TAS2R43* in HGFs and the evident Ca^2+^ evocative effects of their agonists, the potential physiological functions of these TAS2Rs in the periodontium can be expected. Among the five most expressed *TAS2Rs*, TAS2R16 mediated the most robust Ca^2+^ accumulative effects in response to salicin and thus may exert regulatory effects on the physiology of HGFs and periodontal health.

Instead of taste perception, *TAS2Rs*-expressing extra-gustatory cells are immune sentinels in the mammalian innate immune response. TAS2Rs are broadly tuned for bacterial compounds and play a role in nitric oxide production, cilia beating, type 2 immunity initiation, or direct bactericidal effects in the airway and gut (18, 20, 50, 51). In addition, studies have documented the role of TAS2Rs activation in anti-inflammatory effects by suppressing cytokine expression in different cells or tissues (23, 24, 52, 53). Consistently, our study also demonstrated that salicin suppressed the LPS-induced expression of pro-inflammatory cytokines, including IL-6 and IL-8 *via* TAS2R16 activation. Neutrophils constitute the majority of leukocytes recruited to the periodontium. Neutrophils are necessary for maintaining gingival health; nonetheless, they are recently recognized as major players in periodontitis by contributing to substantial tissue destruction (54). The hyperactivation and excessive neutrophil-mediated tissue injury have been detected in aggressive periodontitis and all stages of chronic periodontitis (55–57). HGFs, as the major components of the gingival connective tissue, are transcriptionally active in expressing chemokines and specifically wire toward neutrophils recruitment when encountering infections and damages (5). Among the chemokines that regulate neutrophil migration, IL-8 is the most significant and well-characterized chemotactic and activating factor for neutrophils (58, 59). Here, we also found that salicin weakened LPS-induced neutrophil recruitment in a TAS2R16-dependent manner, likely due to the downregulated release of pro-inflammatory cytokines, particularly IL-8. The suppressed over-activation of cytokine release and over-migration of neutrophils may protect periodontal tissue injury and thus benefit inflammation control.

The NF-κB signaling pathway plays a central role in the LPS-induced expression of cytokines in various cell types (7, 39, 60), and is a major contributor to numerous chronic inflammatory diseases, including periodontitis (61, 62). However, the potential relationship between the taste signaling cascade and the NF-κB pathway has not yet been well documented. Here, we demonstrated that TAS2R16 activation by salicin inhibited the LPS-induced NF-κB cascade by repressing the phosphorylation and nuclear translocation of p65 in HGFs. Activation of TAS2Rs can elevate intracellular calcium levels *via* the PLCβ2-inositol 1,4,5-trisphosphate (IP_3_)-Ca^2+^ axis (9, 22). PLC signaling positively correlates with inflammatory responses in various cell types (63–65), and Plcβ2-deficiency alleviates LPS-induced inflammation and tissue injury (66). In addition, inhibition of PLC signaling and intracellular calcium release can suppress NF-κB translocation (67, 68). Consistently, our study also demonstrated that blocking of PLC and its subsequent Ca^2+^ accumulation by U73122 effectively inhibited pro-inflammatory cytokine expression and NF-κB p65 nuclear translocation induced by LPS. More importantly, we demonstrated that salicin treatment significantly elevated the intracellular level of calcium, and reduced the inflammatory cytokine expression in LPS-induced HGFs. Although LPS-induced cytokine expression is partly dependent on intracellular calcium elevation mediated by PLC (40, 41), LPS *per se* can reduce PLCβ2 expression in a time-dependent manner (69, 70). In addition, our data showed that inhibition of intracellular calcium by U73122 failed to counter the inhibitory effect of salicin on LPS-induced IL-8 expression and p65 nuclear translocation, further suggesting that salicin is less likely to antagonize LPS-induced cytokine expression and NF-κB activation *via* PLCβ2-IP_3_-Ca^2+^ under this context. Other pathway(s) that can be elicited by TAS2Rs activation may contribute to the inhibitory effect of salicin on the inflammatory cytokine expression and NF-κB activation in the LPS-induced HGFs.

Activation of TAS2Rs can cause the separation of the heterotrimeric G-protein subunits into Gα-gustducin and the βγ-gustducin dimer. The βγ-gustducin increases intracellular calcium *via* the PLCβ2-IP_3_-Ca^2+^ axis, while the α-gustducin stimulates phosphodiesterase (PDE) that hydrolyzes cAMP, thereby decreasing intracellular cAMP levels (71, 72). Recent studies have indicated that cAMP positively affects NF-κB activity by targeting the p65/RelA ser^276^ residue through its main effector protein kinase A (PKA) in different cells (37, 38, 73–79). Moreover, cAMP/PKA/cAMP-response element binding protein (CREB) signaling pathway may promote LPS-induced pro-inflammatory cytokine release, including IL-6, IL-33, and TNF-α (80–82). On the other hand, LPS can cause a significant dose and time-dependent increase in forskolin-stimulated adenylate cyclase (AC) activity (83), which in turn elevates intracellular cAMP levels. In the current study, the activation of TAS2R16 by salicin did not decrease the intracellular level of cAMP in the absence of LPS induction, consistent with that reported in other cells (84, 85). Nonetheless, LPS-induction significantly increased cAMP levels in the HGFs, and salicin treatment decreased the LPS-induced cAMP accumulation in a TAS2R16-dependent manner and inhibited the inflammatory cytokine expression and NF-κB activation in this context. In addition, the inhibitory effects of salicin on the LPS-induced NF-κB cascade were countered by elevation of cAMP using forskolin, an AC activator, further suggesting that salicin can activate TAS2R signaling and exert anti-inflammatory effects on the LPS-induced HGFs *via* repressing intracellular cAMP.

Given the wide existence of TAS2Rs and their physiological functions, the application of bitter compounds to the treatment of diseases is possible. TAS2Rs may play a critical role in the pharmaceutical activities of herbal medicines (86). Berberine, a potential bitter agonist for TAS2R38 and TAS2R46 (86), exerts a protective effect in inhibiting inflammatory responses and has a long history in the treatment of inflammatory bowel diseases (87, 88). *Scutellaria baicalensis* Georgi, one of the most widely used herbal medicines, shows anti-inflammatory effects in treating respiratory tract and gut diseases (89). Baicalin, baicalein, and wogonin, the most abundant bioactive components extracted from *Scutellaria baicalensis* Georgi, can activate TAS2R14 as bitter agonists (86, 89, 90). Moreover, our previous study showed that in mice with periodontitis, treatment with the bitter component denatonium benzoate activated gSCCs to produce more antimicrobial peptides and inhibit bacterial colonization, thus alleviating alveolar bone loss in periodontitis (25). Given that TAS2R16 activation inhibited the inflammatory response in HGFs, TAS2Rs may be exploited as a potential target for the treatment of periodontitis. Further studies are warranted to investigate whether TAS2R16 activation can alleviate alveolar bone loss in animal models as well as in clinical cohorts.

Taken together, the current study identified and profiled the expression of *TAS2Rs* in HGFs and discovered calcium signaling in response to various bitter tastants. Furthermore, salicin, a specific agonist of TAS2R16, exerted anti-inflammatory effects in a TAS2R16-dependent manner by inhibiting the cAMP and NF-κB cascade. Thus, our data suggest the possibility of using TAS2Rs as a drug target for the treatment of periodontitis.

## Data Availability Statement

The original contributions presented in the study are included in the article/**Supplementary Material**. Further inquiries can be directed to the corresponding authors.

## Ethics Statement

The studies involving human participants were reviewed and approved by Institution Review Board of West China Hospital of Stomatology, Sichuan University (WCHSIRB-OT-2020-049). The patients/participants provided their written informed consent to participate in this study.

## Author Contributions

ZZ, RX, JXL, and XP carried out the experiments. XX, JYL, XDZ, and XZ conceived the study and provided advice. ZZ, LZ, and RX drafted the manuscript. All authors edited and approved the manuscript.

## Funding

This study was supported by the National Natural Science Foundation of China (81771099, 81870754, 81991500, 81991501, 81900995), a research funding for talents developing, West China Hospital of Stomatology Sichuan University (RCDWJS2020-11), China Postdoctoral Science Founding (2020M673266), and a research grant from the West China School of Stomatology Sichuan University (LCYJ2019-4).

## Conflict of Interest

The authors declare that the research was conducted in the absence of any commercial or financial relationships that could be construed as a potential conflict of interest.

## Publisher’s Note

All claims expressed in this article are solely those of the authors and do not necessarily represent those of their affiliated organizations, or those of the publisher, the editors and the reviewers. Any product that may be evaluated in this article, or claim that may be made by its manufacturer, is not guaranteed or endorsed by the publisher.
