# Supplementary material for: TAS2R16 Activation Suppresses LPS-Induced Cytokine Expression in Human Gingival Fibroblasts
Source: Front Immunol. 2021 Dec 15;12:726546. doi: 10.3389/fimmu.2021.726546 (PMC8714777; doi:10.3389/fimmu.2021.726546)
Supplement: Supplementary file 1 [file DataSheet_1.docx]

Supplementary Material

# Supplementary Data


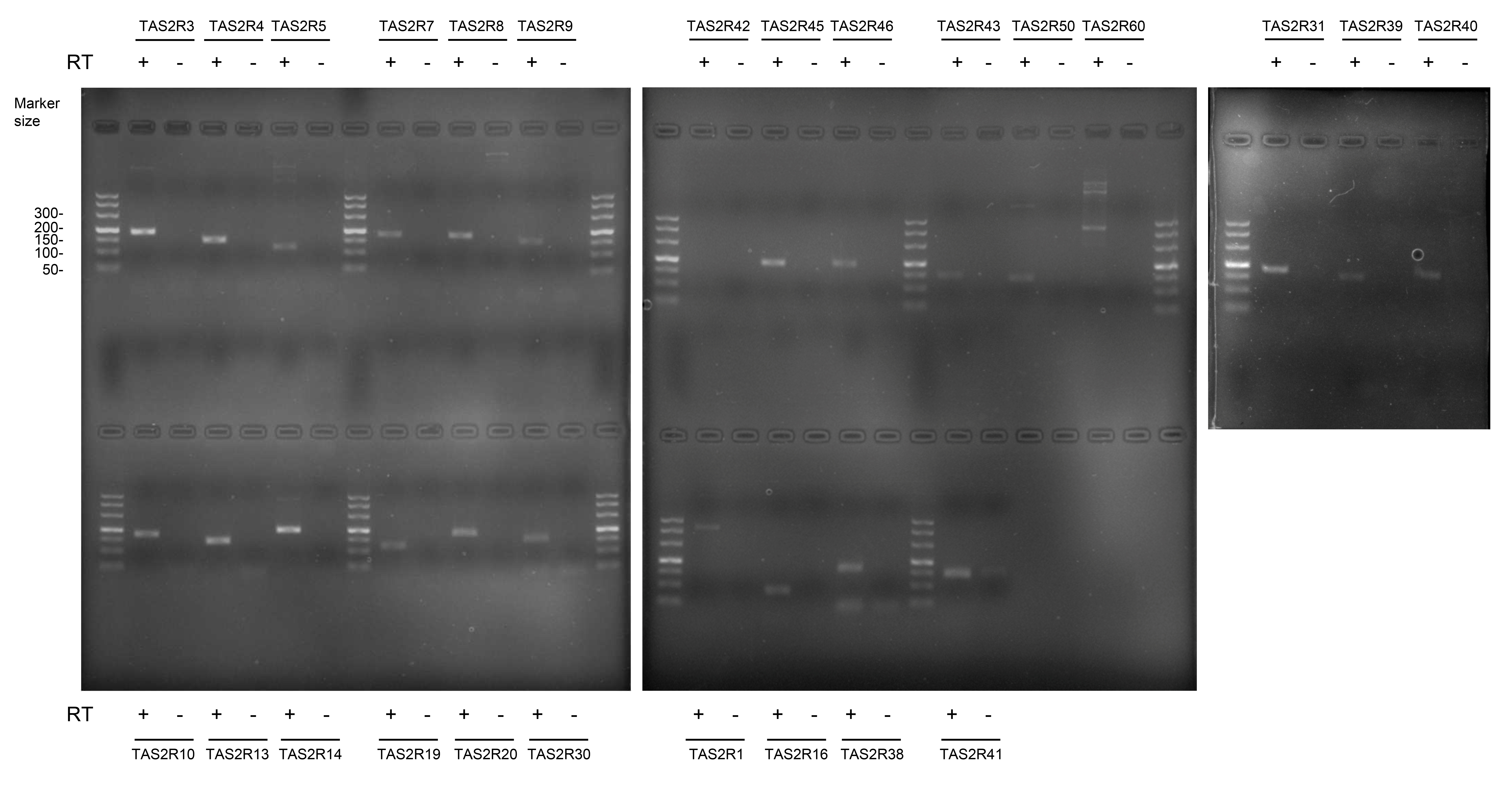


**Supplementary Figure 1.** Expression of *TAS2Rs* in human gingival tissues (pooled from 8 volunteers) examined by RT-PCR. RT +/−: with/without reverse transcription.


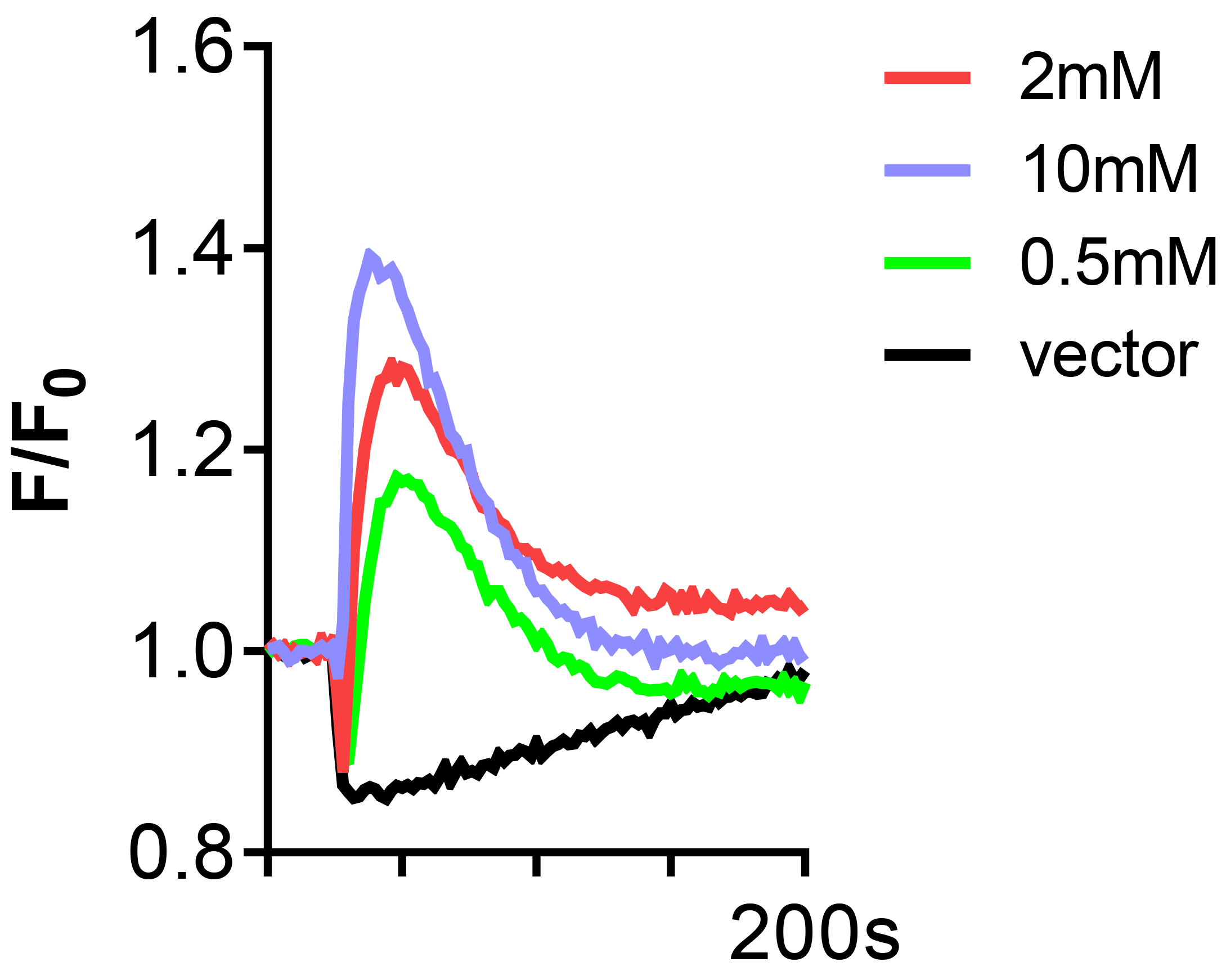


**Supplementary Figure 2.** TAS2R16 could be activated by salicin. Calcium responses of TAS2R16 a nd Gα16gust44-transfected HEK293 cells to indicated concentrations of salicin. Data are normalized by baseline (F/F_0_) and presented as mean from 3 independent experiments. HEK293 transfected with empty vector and Gα16gust44 is severed as the negative control.


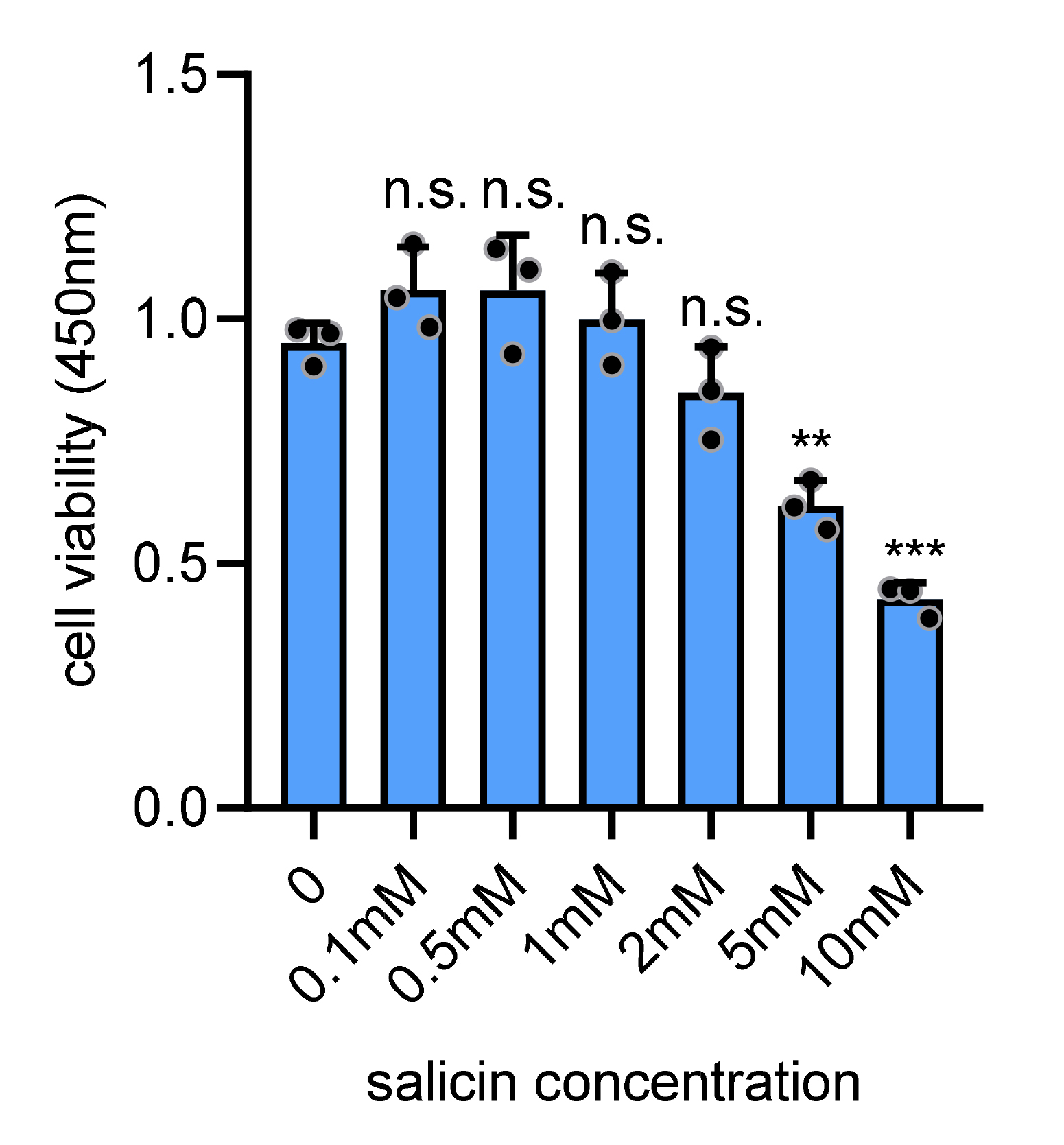


**Supplementary Figure 3.** Cell viability of HGFs exposed to different concentrations of salicin. Data are presented as mean ± s.d.. Each circle represents a datum from one well of cultured cells (n=3). Comparisons between different groups were performed by one-way ANOVA of variance test followed by Tukey’s multiple comparisons test. **: *p*＜0.01; ***: *p*＜0.001; n.s.: not significant.


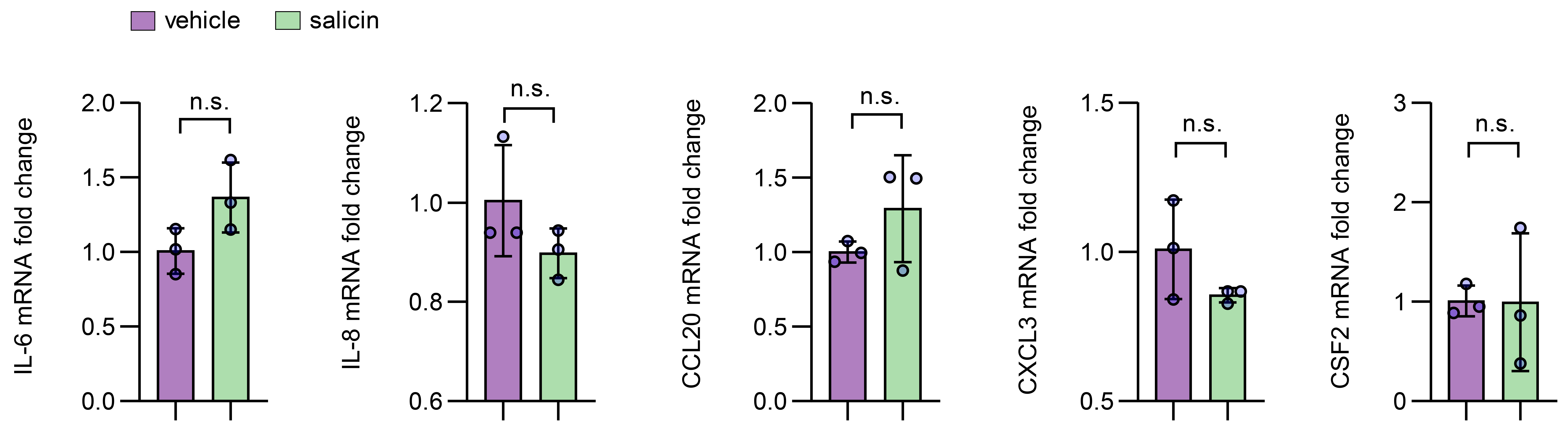


**Supplementary Figure 4.** Salicin treatment does not alter cytokine expression of HGFs in the absence of LPS-induced inflammation. mRNA expression levels of cytokines in HGFs treated with salicin (2 mM) or vehicle are determined by RT-qPCR. The expression level of each target is normalized with *GAPDH*. Data are represented as mean ± s.d.. Each circle represents a datum from one sample (n=3). The comparison was performed by unpaired t-test. n.s.: not significant.


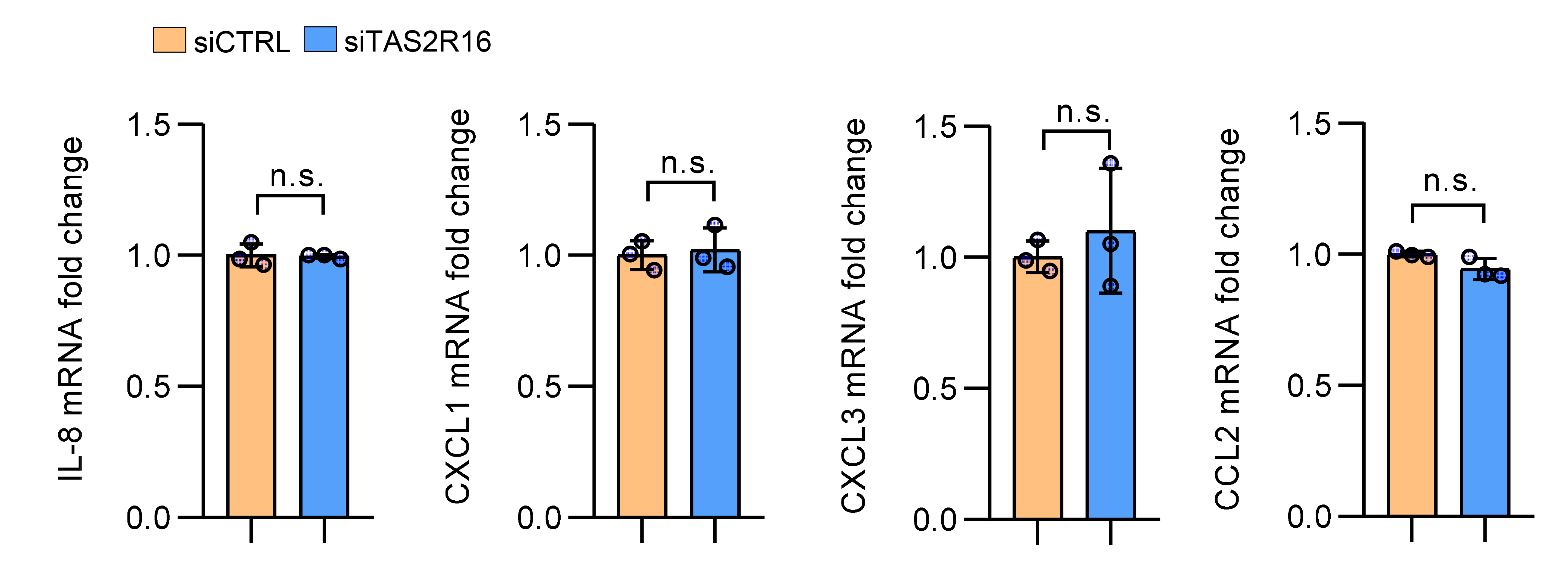


**Supplementary Figure 5.** TAS2R16 knock-down does not alter selected cytokine expression of HGFs in the absence of LPS-induced inflammation. The expression level of each target is normalized with *GAPDH*. Data are represented as mean ± s.d.. Each circle represents a datum from one sample (n=3). The comparison was performed by unpaired t-test. n.s.: not significant.


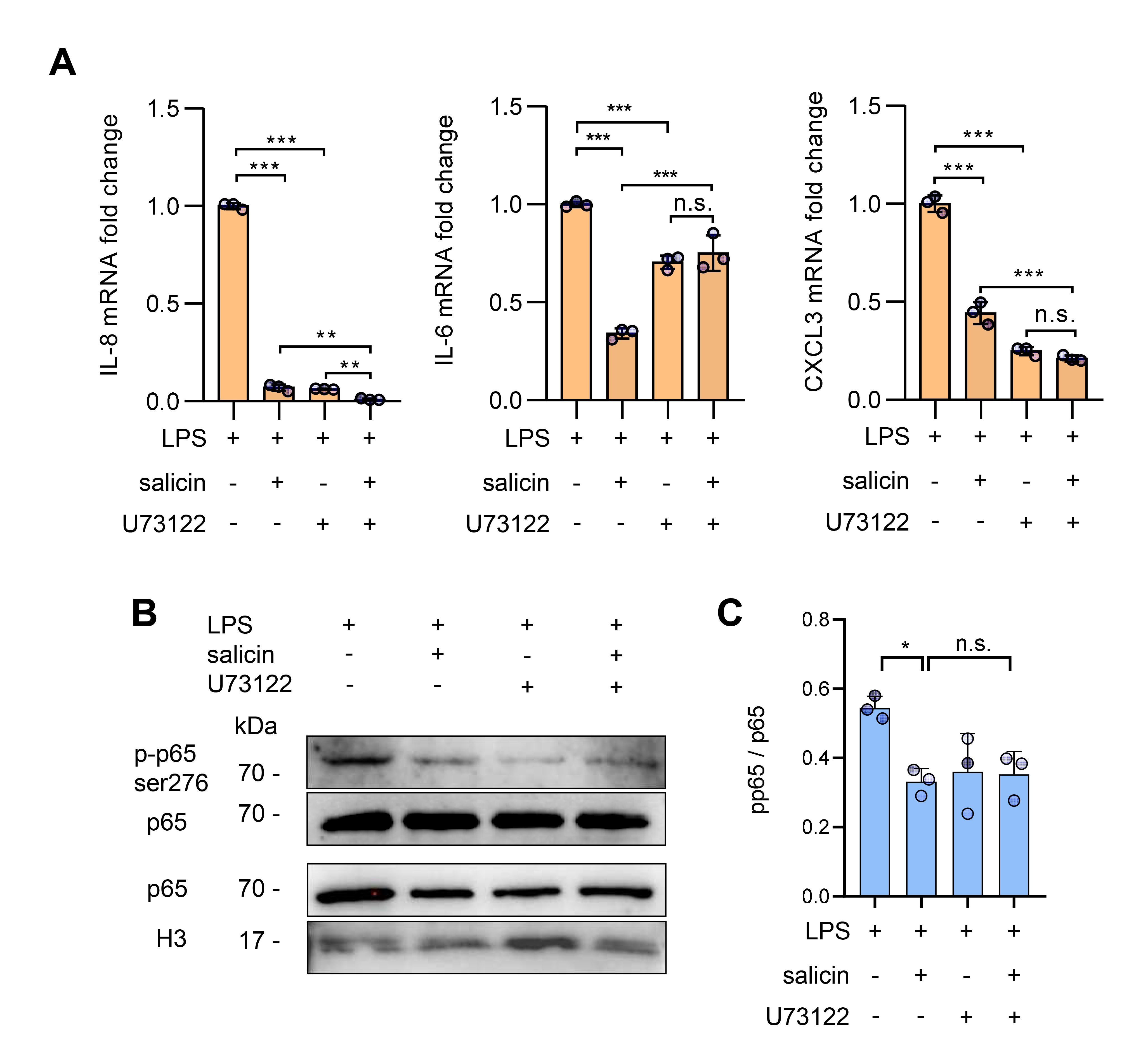


**Supplementary Figure 6.** Treatment of U73122 significantly inhibits LPS-induced cytokine expression and NF-κB activation. **(A)** Cytokine mRNA expression level of HGFs determined by RT-qPCR. The expression level of each target is normalized with *GAPDH*. Each circle represents a datum from one sample (n=3). **(B)** Representative Western blot images for p65 phosphorylation and nuclear translocation from HGFs treated with LPS, salicin, and U73122. **(C)** The normalized ratio of p65-ser^276^ to p65. Each circle represents an independent experiment. LPS, 5 μg/mL; salicin, 2 mM; U73122, 0.5 μM. Data are represented as mean ± s.d.. Comparisons between different groups were performed by one-way ANOVA of variance test followed by Tukey’s multiple comparisons test. *: *p*＜0.05; **: *p*＜0.01; ***: *p*＜0.001; n.s.: not significant.

**Supplementary Table 1. Primer and siRNA list.**

| **Targets** | **sequence (5’-3’)** |
| --- | --- |
| GNAT3 | F-AGAGGACCAACGACAACTTTATG |
|  | R-AGCCGTTTTATTACCTCAGCC |
| TRPM4 | F-GCACGACGTTCATAGTTGACT |
|  | R-CTTCTCCGTGGTGTGTGCAT |
| TRPM5 | F-CTGCGGCTGATCCATATCTTT |
|  | R-TCCTTCATCATGCGCTCTACC |
| PLCB2 | F-GCCCAGGAGAGGTTGAAGAG |
|  | R-GCCTCCTTCTGGAACTGCTT |
| TAS2R1 | F-CAGAAAAATGGCTCCGCTGG |
|  | R-CCATGTGGCAAGCCAAAGTT |
| TAS2R3 | F-TGGCACTCTTGAGGATCATTCT |
|  | R-CTGGCGATTTTCAGGCAGTAG |
| TAS2R4 | F-TTCCTGAACTTGTGACTACGAGA |
|  | R-CCTCAAGGAGTGTATTAGCAAGG |
| TAS2R5 | F-CTGGGAGACACTCATGGCAG |
|  | R-CTCCGAGCACACACTGTCTT |
| TAS2R7 | F-CCTGGATTCTACTGGGGTGC |
|  | R-GCGTTGCCAGGTTGAGAAAT |
| TAS2R8 | F-AACTCTATGCTACCGGCAGTA |
|  | R-TGCTGCAATCTCTCCAAACTC |
| TAS2R9 | F-ATAGCGTGCTAGTAAGCATTGTG |
|  | R-TTGTTGATCTTTAGCTTCAGCCA |
| TAS2R10 | F-TGGCTTTATTCTCACCGGCT |
|  | R-GCTGGTGGCAAACCACATAC |
| TAS2R13 | F-GAAAGTGCCCTGCCGAGTATC |
|  | R-AGGAGTTTATCGACTGAGGACA |
| TAS2R14 | F-TTCGGTTGATCGGATCCTCAC |
|  | R-AAAAGTACCGAGGCCTGTAGC |
| TAS2R16 | F-GGCCTCCACCATCTTTCTCAT |
|  | R-CGCGCTTTCATGCTTGGATT |
| TAS2R19 | F-CTGGTGGTCTCCAGAATTGGT |
|  | R-ACAGCCCAGGCATTAGAAGC |
| TAS2R20 | F-AGCTCTGCAAACTGTGACCT |
|  | R-AGCGTCTTGTTCCCCCAAAT |
| TAS2R30 | F-ATTTTGGGAGGCTGGAAAAGC |
|  | R-TTCACCCAGTACCTCACATGC |
| TAS2R31 | F-AACCACGCTAGGAAACTTAGTGC |
|  | R-ACGGCACATAACAAGAGGAAAA |
| TAS2R38 | F-TGTTCCTGAGTGCTATCCAGC |
|  | R-GGTGTGAGAGAAACGGATGAG |
| TAS2R39 | F-TGCGATCCTGCAGAAAGTGA |
|  | R-CTGCCACTTGTGGAAACTGC |
| TAS2R40 | F-CAACGGTGAACACAGATGCC |
|  | R-GTCACCAGTGGGGAGTGTTT |
| TAS2R41 | F-CCTCATTAGCTTGGGTGCCT |
|  | R-GCTGAGTTCAGGAAGTGCCA |
| TAS2R42 | F-GCTGGGGAATGTGTTCATTGG |
|  | R-GTGGAGATAGCCAAGCAGGT |
| TAS2R43 | F-TAATATCTGGGCAGTGATCAACC |
|  | R-CCCAACAACATCACCAGAATGA |
| TAS2R45 | F-CACCGAGTGGGTGAAGAGAC |
|  | R-TGCTGAAATGGCCGGTTACT |
| TAS2R46 | F-GCAATGTACCTTTCAAATACAACG |
|  | R-GGAAGGAGGTCACAGTTTGC |
| TAS2R50 | F-TCGCAAGATCTCAGCACCAA |
|  | R-ATGACAACCGGGTCATTCCG |
| TAS2R60 | F-GGTGTTCAGTGCTGCAGGTA |
|  | R-CACACCTTGAGGAACGACGA |
| IL1A | F-TGGTAGTAGCAACCAACGGGA |
|  | R-ACTTTGATTGAGGGCGTCATTC |
| IL1B | F-AGCTACGAATCTCCGACCAC |
|  | R-CGTTATCCCATGTGTCGAAGAA |
| IL6 | F- AATCATCACTGGTCTTTTGGAG |
|  | R- GCATTTGTGGTTGGGTCA |
| IL7 | F-TTCCTCCCCTGATCCTTGTTC |
|  | R-CTTGCGAGCAGCACGGAATA |
| IL8 | F-GACATACTCCAAACCTTTCCACC |
|  | R- AACTTCTCCACAACCCTCTGC |
| IL12A | F-ATGGCCCTGTGCCTTAGTAGT |
|  | R-AGCTTTGCATTCATGGTCTTGA |
| IL32 | F-TGGCGGCTTATTATGAGGAGC |
|  | R-CTCGGCACCGTAATCCATCTC |
| IL33 | F-GTGACGGTGTTGATGGTAAGAT |
|  | R-AGCTCCACAGAGTGTTCCTTG |
| TNF | F-GGGCTCCAGGCGGTGCTTGTTC |
|  | R-CAGGCTTGTCACTCGGGGTTCG |
| CXCL1 | F-CAGGGAATTCACCCCAAGAACA |
|  | R-GGATGCAGGATTGAGGCAAGC |
| CXCL2 | F-CTCAAGAATGGGCAGAAAGC |
|  | R-AAACACATTAGGCGCAATCC |
| CXCL3 | F-CCAAACCGAAGTCATAGCCAC |
|  | R-TGCTCCCCTTGTTCAGTATCT |
| CXCL5 | F-AGCTGCGTTGCGTTTGTTTAC |
|  | R-TGGCGAACACTTGCAGATTAC |
| CXCL12 | F-ATTCTCAACACTCCAAACTGTGC |
|  | R-ACTTTAGCTTCGGGTCAATGC |
| CXCL16 | F-CCCGCCATCGGTTCAGTTC |
|  | R-CCCCGAGTAAGCATGTCCAC |
| CCL2 | F-AATCAATGCCCCAGTCACCT |
|  | R-GGGTCAGCACAGATCTCCTT |
| CCL7 | F-CCACACAGAAGTGGGTCCAG |
|  | R-ACCACTCTGAGAAAGGACAGG |
| CCL20 | F-TTTATTGTGGGCTTCACACG |
|  | R-GATTTGCGCACACAGACAAC |
| LIF | F-CCAACGTGACGGACTTCCC |
|  | R-TACACGACTATGCGGTACAGC |
| CSF1 | F-TGGCGAGCAGGAGTATCAC |
|  | R-AGGTCTCCATCTGACTGTCAAT |
| CSF2 | F-TCCTGAACCTGAGTAGAGACAC |
|  | R-TGCTGCTTGTAGTGGCTGG |
| GAPDH | F-GGAGCGAGATCCCTCCAAAAT |
|  | R-GGCTGTTGTCATACTTCTCATGG |
| si-TAS2R16 sense  si-TAS2R16 antisense  non-target si-Ctrl sense | CCAUCCAACUCACUGUCUUTT  AAGACAGUGAGUUGGAUGGGT  GUGAGCGUCUAUAUACCAUTT |
| non-target si-Ctrl antisense | AUGGUAUAUAGACGCUCACTT |

**Supplementary Table 2. Bitter components used in the current study.**

| **Bitter tastants** | **Receptors** |
| --- | --- |
| acesulfame K | TAS2R43, TAS2R44 |
| acetaminophen | TAS2R39 |
| aristolochic acid | TAS2R31, TAS2R43, TAS2R44 |
| camphor  chloramphenicol | TAS2R4, TAS2R10, TAS2R14  TAS2R1, TAS2R8, TAS2R10, TAS2R39, TAS2R43, TAS2R46 |
| chloroquine | TAS2R3, TAS2R10, TAS2R39 |
| chlorpheniramine | TAS2R4, TAS2R7, TAS2R10, TAS2R14, TAS2R38, TAS2R39, TAS2R40, TAS2R46 |
| denatonium benzoate | TAS2R4, TAS2R8, TAS2R10, TAS2R13, TAS2R39, TAS2R43, TAS2R46 |
| EGCG | TAS2R39, TAS2R14 |
| PTC | TAS2R38 |
| quinine | TAS2R4, TAS2R7, TAS2R10, TAS2R14, TAS2R39, TAS2R40, TAS2R43, TAS2R44, TAS2R46 |
| D (-)-salicin | TAS2R16 |
| Sodium cyclamate | TAS2R1, TAS2R38 |
| thiamine | TAS2R1, TAS2R39 |

**Supplementary Table 3. Gene Expression Omnibus datasets for expression analysis of *TAS1Rs*, *TAS2Rs* and downstream elements in primary gingival fibroblasts.**

| Accession number | *TAS1Rs* expression | *TAS2Rs* expression | Downstream components expression |
| --- | --- | --- | --- |
| GDS5607 (1) | *TAS1R1, TAS1R2* | *TAS2R1, TAS2R3, TAS2R4, TAS2R5, TAS2R7, TAS2R8, TAS2R9, TAS2R10, TAS2R13, TAS2R14, TAS2R16, TAS2R19, TAS2R38, TAS2R39, TAS2R40, TAS2R41, TAS2R45, TAS2R50* | *GNAT3, PLCB2, TRPM5, TRPM4* |
| GDS1685 (2) | *TAS1R1, TAS1R2* | *TAS2R1, TAS2R3, TAS2R4, TAS2R5, TAS2R7, TAS2R8, TAS2R9, TAS2R10, TAS2R13, TAS2R14, TAS2R16, TAS2R19, TAS2R38, TAS2R39, TAS2R40, TAS2R41, TAS2R45, TAS2R50* | *GNAT3, PLCB2, TRPM5, TRPM4* |
| GDS5811 (3) | *TAS1R1, TAS1R2, TAS1R3* | *Except for TAS2R30* | *PLCB2, TRPM5, TRPM4* |
| GSM2179385 (4) | *TAS1R1, TAS1R3* | *TAS2R8, TAS2R10, TAS2R13, TAS2R14, TAS2R19, TAS2R20, TAS2R30, TAS2R31, TAS2R40, TAS2R43, TAS2R46, TAS2R50, TAS2R60* | *PLCB2, TRPM5, TRPM4* |
| GSE68685 (5) | *TAS1R1, TAS1R2, TAS1R3* | *All 25 TAS2Rs* | *PLCB2, TRPM5, TRPM4* |
| GSE140523 (6) | *TAS1R1,*  *TAS1R3* | *TAS2R1, TAS2R3, TAS2R4, TAS2R5, TAS2R8, TAS2R9, TAS2R10, TAS2R13, TAS2R14, TAS2R19, TAS2R20, TAS2R30, TAS2R31, TAS2R43, TAS2R46, TAS2R50* | *GNAT3, PLCB2, TRPM5, TRPM4* |

**Supplementary Table 4. Expression profiling of TAS2Rs downstream machineries from selected datasets in** **Gene Expression Omnibus datasets**

|  | *GNAT3* | *PLCB2* | *TRPM4* | *TRPM5* |
| --- | --- | --- | --- | --- |
| GDS5607 | 67.86 | 86.58 | 93.87 | 12.54 |
| GDS1685 | 5.70 | 17.80 | 79.13 | 5.23 |
| GDS5811* |  | 5.84 | 8.69 | 6.87 |
| GSM2179385* |  | 6.25 | 8.17 | 6.13 |
| GSE68685* |  | 6.13 | 8.17 | 6.25 |
| GSE140523 | 0.80 | 5.89 | 296.20 | 0.20 |

*, Data exhibited in the Gene Expression Omnibus datasets are transformed counts.

**Supplementary Table 5. mRNA expression of selected cytokines in HGFs treated with LPS or vehicle (normalized by *GAPDH*).**

| gene | vehicle-treatment | LPS-treatment | *P* value |
| --- | --- | --- | --- |
| IL8 | 0.43 | 3.66 | <0.0001 |
| CCL2 | 0.25 | 0.53 | 0.0015 |
| CXCL1 | 0.13 | 0.36 | 0.0001 |
| IL6 | 0.049 | 0.28 | 0.0001 |
| CXCL3 | 0.024 | 0.13 | <0.0001 |
| CXCL12 | 0.13 | 0.037 | <0.0001 |
| CCL20 | 0.0016 | 0.016 | <0.0001 |
| CXCL5 | 0.0019 | 0.015 | <0.0001 |
| CXCL16 | 0.012 | 0.014 | 0.0002 |
| CSF1 | 0.017 | 0.014 | 0.0071 |
| LIF | 0.014 | 0.012 | 0.0891 |
| CSF2 | 0.00041 | 0.0097 | 0.0002 |
| IL1B | 0.00031 | 0.0083 | <0.0001 |
| CCL7 | 0.0021 | 0.0054 | 0.0097 |
| IL32 | 0.0016 | 0.0029 | <0.0001 |
| IL12A | 0.00053 | 0.0017 | <0.0001 |
| CXCL2 | 0.000032 | 0.00029 | 0.0198 |
| IL33 | 0.000024 | 0.00025 | <0.0001 |
| IL7 | 0.000035 | 0.000044 | 0.083 |
| IFNE | 0.000060 | 0.000039 | 0.0056 |
| IL1A | None detected | 0.000029 |  |
| TNFA | 0.000051 | 0.000021 | <0.0001 |

**Reference**

1. Lappin-Scott H, Burton S, Stoodley P. Revealing a world of biofilms--the pioneering research of Bill Costerton. *Nat Rev Microbiol* (2014) 12(11):781-7. doi: 10.1038/nrmicro3343.

2. Zhu Y, Zhang W, Huo Z, Zhang Y, Xia Y, Li B, et al. A novel locus for maternally inherited human gingival fibromatosis at chromosome 11p15. *Hum Genet* (2007) 121(1):113-23. doi: 10.1007/s00439-006-0283-1.

3. Kuk H, Hutchenreuther J, Murphy-Marshman H, Carter D, Leask A. 5Z-7-Oxozeanol Inhibits the Effects of TGFbeta1 on Human Gingival Fibroblasts. *PLoS One* (2015) 10(4):e0123689. doi: 10.1371/journal.pone.0123689.

4. Horie M, Yamaguchi Y, Saito A, Nagase T, Lizio M, Itoh M, et al. Transcriptome analysis of periodontitis-associated fibroblasts by CAGE sequencing identified DLX5 and RUNX2 long variant as novel regulators involved in periodontitis. *Sci Rep* (2016) 6:33666. doi: 10.1038/srep33666.

5. Williams RC, Skelton AJ, Todryk SM, Rowan AD, Preshaw PM, Taylor JJ. Leptin and Pro-Inflammatory Stimuli Synergistically Upregulate MMP-1 and MMP-3 Secretion in Human Gingival Fibroblasts. *PLoS One* (2016) 11(2):e0148024. doi: 10.1371/journal.pone.0148024.

6. Foote AG, Wang Z, Kendziorski C, Thibeault SL. Tissue specific human fibroblast differential expression based on RNAsequencing analysis. *BMC Genomics* (2019) 20(1):308. doi: 10.1186/s12864-019-5682-5.
